# The Biology and Control of Leishmaniasis Vectors

**DOI:** 10.4103/0974-777X.62866

**Published:** 2010

**Authors:** David M Claborn

**Affiliations:** *Department of Nursing, Missouri State University, Springfield, MO, USA*

**Keywords:** Leishmaniasis, *Lutzomyia*, Phlebotomine, *Phlebotomus*, Sand fly

## Abstract

Vector control remains a key component of many anti-leishmaniasis programs and probably will remain so until an effective vaccine becomes available. Technologies similar to those used for control of adult mosquitoes, specifically interior residual sprays and insecticide-treated nets, are currently at the forefront as disease control measures. This article provides a review of literature on the biology and control of sand fly vectors of leishmaniasis in the context of changing disease risks and the realities of modern vector control. The Literature Retrieval System of the Armed Forces Pest Management Board, Washington, DC, was the primary search engine used to review the literature.

Leishmaniasis is an emerging disease with an ability to adapt to changing environments. Ongoing epidemics and the military importance of the disease, along with the lack of a vaccine or chemoprophylaxis, have increased interest in the biology and control of the disease vectors: phlebotomine sand flies. This review addresses the biology and control of the sand fly, as well as aspects of the disease and parasite biology that impact the control of the disease vectors. It emphasizes recent literature but includes historical references to regional disease vectors as well. Literature searches were performed on two systems. The PUBMED system provided through the National Library of Medicine website was used to review the immunology, parasitology and treatment of leishmaniasis. The Literature Retrieval System of the Armed Forces Pest Management Board, which includes the Norman Gratz collection, was used to obtain articles on the biology and control of leishmaniasis vectors and the epidemiology of the disease. Articles were ranked by date in order to emphasize recent literature; however, important historical literature and reviews were included. Note that the term “sand fly” is used throughout this review, though “sandfly” may be used in quoted or cited material.

## LITERATURE REVIEW

Numerous factors have contributed to increased global interest in the epidemiology, control and treatment of leishmaniasis. This vector-borne disease is an emerging infection that is adapting to changing environments and spreading into new geographical regions. The physical effects of leishmaniasis can range from mild scarring to grotesque disfiguration and death, so its presence in a community is often very obvious and disturbing. Foremost among the factors causing increased interest is the persistent and deadly epidemic of visceral leishmaniasis in Sudan.[1] Incidence rates in eastern Sudan were 20 to 38 cases per 1,000 person-years between 1991 and 1996, prompting the establishment of multiple leishmaniasis treatment centers by nongovernmental organizations.[2] These centers were quickly overwhelmed. The recent expulsion of the aid agencies by the Sudanese government will undoubtedly further hamper efforts to prevent and treat this disease. The degree to which the expulsion will increase mortality rates is unknown; however, the 1984-1994 epidemic is estimated to have killed about one third of the population of the western Upper Nile province, an estimated death total of nearly 100,000 people.[3]

Another factor which has increased interest in leishmaniasis is the impact of the disease on military operations in the Middle East and Afghanistan, particularly the operations of the United States. Even though leishmaniasis caused few illnesses in the 1991 Operation Desert Storm, an unusual manifestation similar to the visceral form of the disease threatened military blood supplies and led to postdeployment diagnostic problems.[4] The “viscerotropic” disease occurred when a parasite species normally associated with the less dangerous cutaneous disease (*Leishmania tropica)* displayed symptoms similar to those of the more deadly visceral leishmaniasis.[5] More recent military operations in Iraq and Afghanistan have produced many more cases of the disease. Between 2001 and 2006, nearly 1,300 cases of leishmaniasis were diagnosed in American military personnel returning from war zones, including Afghanistan.[6] Peak incidence rates were observed in late summer of 2003 but have declined since then. Nevertheless, leishmaniasis continues to be a disease of great interest to militaries, especially those which operate internationally.

Surprising developments in canine leishmaniasis have also contributed to increased interest in leishmaniasis. The geographic distribution of canine disease appears to have spread from the warmer climate of Sicily and the Mediterranean to northern Italy[7] and other places in Europe. The importance of canine leishmaniasis as a possible measure of human disease risk was demonstrated in Brazil, where a rise in the number of human cases was preceded by an increase in the prevalence of canine disease.[8] Although the majority of infected dogs are asymptomatic carriers, there is some concern that the dogs that show no sign of disease can potentially spread the disease. Therefore, there can be little surprise at the alarm that accompanied discovery of *Leishmania infantum* in foxhound populations in the eastern United States. Infected dogs were found as far north as New York.[9] Although there are some questions as to the importance of sand flies as vectors of canine leishmaniasis in foxhound kennels, sand flies were collected in the vicinity of infected dogs. This was the first reported collection of this sand fly from New York. The species collected (*Lutzomyia vexator*) is not known to be a vector of the parasite that causes leishmaniasis in dogs or humans; it reportedly feeds primarily on cold-blooded animals.[10] Nevertheless, the distributions of the parasite and the fly are both more extensive than previously thought.

These factors and others have increased interest in leishmaniasis and its vectors. The lack of an effective vaccine, and the expense, side effects and difficulties associated with treatment of the disease, serve to emphasize the importance of vector control in disease prevention.

## THE PARASITE AND CLINICAL DISEASE

Leishmaniasis is the result of parasitic infection with a protozoan flagellate of the family Trypanosomatidae, order Kinetoplastida.[11] The genus is *Leishmania.* The parasite exhibits two primary stages in its development: the amastigote and the promastigote. The amastigote infects lisosomal vacuoles in the phagocytic cells of a vertebrate host; whereas, the promastigote is an extracellular form which attaches to the intestinal microvilli of the insect midgut (or may be found free). Thus *Leishmania* are obligatory parasites that alternate between the invertebrate vector (the sand fly) and a vertebrate host, which may be human, canine, rodent or other. The female sand fly acquires the infection while taking a blood meal from the vertebrate host.

Leishmaniasis is probably second only to malaria in importance as a protozoan disease causing human suffering.[12] At least 23 species of *Leishmania* cause human disease, and most occur in the tropics and subtropics of both the Old and New Worlds. Human disease is usually described as occurring in three primary manifestations: cutaneous, mucocutaneous and visceral. Other manifestations have been described as leishmaniasis recidivans, diffuse cutaneous leishmaniasis and post-*kala-azar* dermal leishmaniasis. These latter forms are described in depth elsewhere.[12–14]

Localized cutaneous leishmaniasis (LCL) usually affects uncovered body parts where the sand fly has access to bare skin. At least three parasite species are associated with LCL: *Le. braziliensis* and *Le. mexicana* in the New World, and *Le. major* in the Old World.[14] New World LCL usually manifests as a solitary primary lesion, but Old World disease often presents with multiple primary lesions. The incubation period ranges from about 7 to 90 days. The initial red papule lesion often develops into a circumscribed ulcer with a granulomatous base and hypertrophic borders. Although pain and itching may be mild, secondary infections may occur. The ulcer will usually regress after 6 to 12 months, leaving pigmentation scars. Immunity is not complete, so secondary infections may occur.[14] Diffuse cutaneous leishmaniasis (DCL) occurs when the lesions are disseminated and may resemble lepromatous leprosy. DCL is most frequent with infections by *L. mexicana amazonensis.* Relapses and chronic infections are common.

Mucocutaneous leishmaniasis (MCL) is usually associated with infections by the New World's *Le. brazilensis braziliensis.* The infection often develops initially in the nasal septum and can result in severe mutilation of the lip, gums, tonsils, pharynx and palate. Bony structures are not affected. Damage may be severe enough to cause death through malnutrition and acute respiratory pneumonia.[12,14]

Visceral leishmaniasis (VL), or *kala-azar*, is an infection of the reticuloendothelial system, usually with *Le. donovani* and *Le. infantum*, both Old World species of the parasite; or *Le. chagasi*, a New World species. Other parasite species, including *Le. amazonensis* and *Le. tropica*, have reportedly been involved.[15] Most fatalities due to leishmaniasis are associated with visceral disease, as victims progress through fevers, malaise and weight loss associated with anemia, hepatomegaly and splenomegaly. Secondary bacterial infections leading to tuberculosis, pneumonia and diarrhea contribute to high mortality in untreated disease.

Until the early 1990s, pentavalent antimony was the only first-line drug with a well-documented record of success in the treatment of visceral leishmaniasis.[16] Such treatment required extended hospitalization and could cause severe side effects like pancreatitis and cardiac abnormalities. Now, however, there is a range of treatment options. In Italy, the antimonials have been largely replaced with amphotericin B.[17] Similar changes in treatment have occurred elsewhere. Amphotericin B for treatment of cutaneous leishmaniasis in the United States, however, is largely reserved for antimony failures.[18] Other treatments include oral antifungals and cryotherapy.[18] There is some concern that parasites can persist for the entire life of the patient; thus decline of the immune system with advancing age or immune compromise can result in disease re-emergence. “Cured” patients have been reported to become parasite positive again 1-30 years after treatment.[19] Vaccine development continues, but at present, no effective vaccine is commercially available. Therefore, prevention of exposure to infected sand flies is a primary means of interrupting disease transmission.

## THE VECTOR

The only known vector of the leishmaniase s is the small dipteran fly known commonly as a “sand fly.” The common name can be misleading as other nonvectors are referred to by the same name in certain locales. The vector shares the family Psychodidae with the nonvector, nonbiting moth flies, often seen around shower drains. The subfamily Phlebotominae is comprised of the bloodsucking sand fly vectors of leishmaniasis and other diseases, including bartonellosis (Carrion's disease), phlebotomus fever (sand fly fever) and vesicular stomatitis.[20] Like all true flies (Order: Diptera), sand flies undergo complete metamorphosis and exhibit four complete life stages: egg, larva, pupa and the adult. Unlike mosquitoes, the immature stages do not require standing water to complete development, though they do require relatively warm, moist environments. These requirements are often provided by animal burrows, so the sand flies are frequently found near rodent habitations.

Sand fly eggs are laid in a suitable habitat by the female adults. They are initially white or light gray in color but often turn dark brown or black within a few hours of oviposition, depending on the species. They are banana-shaped and nearly microscopic in size (0.3-0.5 mm in length). Time-to-hatch is highly temperature dependent but averages 6-17 days. The eggs are usually laid in a mass of high organic content, like animal excreta and soil, providing the newly emerged larvae with shelter, moisture and nutrition.

Larvae are caterpillar-shaped with head capsules and small leaf-like antennae. Distinctive caudal setae can help identify the larvae as sand flies, but larvae are rarely used in taxonomy because very few are ever collected in nature.[12] There are four larval instars ranging in size from 0.55 mm long in the 1^st^ to about 3.2 mm long in the 4^th^. The 1^st^ instar larvae usually have two long caudal setae, but the 2^nd^ instar larvae have 4 caudal setae upon molting. The larvae move very little distance from the oviposition site.

Pupae resemble a small butterfly chrysalis except that the 4^th^-stage larval exuvium (cast-off exoskeleton) is attached at one end. The exuvium acts as a glue which is attached to a solid substrate and holds the pupa upright.[12]

Adult sand flies are about one-third the length of a small mosquito, usually less than 3.5 mm in length. They are covered with dense hairs and hold their wings in a characteristic “V” shape over their backs when at rest. The wing veins are parallel to each other and have numerous small “hairs.” The eyes are large and dark. The antennae are long and filiform, with 16 segments. Mouthparts are short, dagger-shaped and oriented downward. The thorax is distinctively humped, pushing the head below the upper surface of the thorax. The legs are very long and delicate.

Both female and male sand fly adults obtain carbohydrate nutrition from plant juices; however, most females also require at least one blood meal in order to complete development of egg batches. Some are autogenous (able to produce viable eggs without a blood meal).[21] Acquisition of disease agents is therefore incidental to blood meals.

Sand flies are very susceptible to dehydration, so most are nocturnal. They seek shelter in animal burrows, tree buttresses or holes, caves, rocks and other protected habitats, including human habitations. Generally weak flyers, they usually fly close to the ground in short hops.[22] Their range is typically very short (about 300 m), but some have been known to travel up to 2300 m in desert environments. The short flight range usually restricts the adult to the general vicinity around the larval development site. These sites are usually organically rich, moist soils. In the New World, the flies are often found near tree buttresses and caves.[23] In the United States, *Lutzomyia shannoni* was found associated with hardwood forests and the ecotonal transitions from hardwood to meadow. Adult flies were more rarely collected in evergreen forest or agricultural fields.[24,25] In more arid environments, such as much of their range in the Old World, sand flies are often associated with contaminated soils of domesticated animal shelters, termite mounds and rodent burrows. They may also occur in the earthen floors of human habitations.[23]

Over 500 species of sand flies have been described from various parts of the world, with most being assigned to three genera: *Phlebotomus* and *Sergentomyia* of the Old World, and *Lutzomyia* of the New World. Vectors in the Old World come primarily from the genus *Phlebotomus*; and in the New World, exclusively from *Lutzomyia.* Only a few dozen species are known to be vectors of human disease.[26] Though several species, and even other genera, have been suggested as potential vectors, the accepted criteria for incriminating vectors are rigorous and difficult to satisfy. Those criteria are as follows:

1. The sand fly species must feed on humans and the reservoir host.
2. The disease agent must be repeatedly isolated from wild-caught sand flies.
3. The sand fly must occur in the geographical location where the disease occurs.
4. The sand fly must support the complete development of the parasite.
5. The sand fly must be able to transmit the parasite to a susceptible host while taking a blood meal.[27]

Table 1 lists several species that have been implicated as vectors of leishmaniasis in various countries. In some cases, the above-mentioned criteria have not been satisfied and the researchers have relied primarily on epidemiological or historical data for implication of the species.

**Table 1:** Some sand fly species that have been reported as vectors or potential vectors in various countries

Region/country	Vector spp.	Disease type	Reference
Argentina/Mexico	*Lutzomyia longipalpis*	Cutaneous/visceral	59
Belize/Mexico	*L. olmeca*	Cutaneous	60
Panama	*L. panamensis*	Cutaneous	61
Brazil	*L. whitmani/intermedia*	Cutaneous	62
Columbia	*L. evansi, gomezi*	Urban	63
Venezuela	*L. vallesi, gomezi*	Cutaneous	64
Sudan	*Phlebotomus langeroni orientalis*	Visceral	65
Kenya/Ethiopia	*P. martini*	Visceral	66
Palestinian W. Bank	*P. papatasi*	Cutaneous	67
	*P. sergentii*	Cutaneous	67
	*P. syriacus*	Visceral	67
N. W. Africa	*P. dubosqi*	Cutaneous	68
Greece	*P. neglectus*	Visceral	69
India	*P. papatasi*	Cutaneous	59
India	*P. argentipes*	Visceral	59
Saudi Arabia	*P. papatasi*	Cutaneous	70
Monaco	*P. perniciosus*	Visceral	71
	*P. ariasi*	Visceral	71
Egypt	*P. langeroni*	Visceral	72
China	*P. alexandri*	Visceral	73
	*P. chinensis*	Visceral	73
	*P. longiductus,*	Visceral	73

## ROLE OF VECTOR CONTROL IN PREVENTION OF LEISHMANIASIS

The lack of a vaccine or chemoprophylaxis limits the options for prevention of leishmaniasis. Primary available tools include (1) elimination of reservoir populations and (2) some form of vector control, including barriers to sand fly feeding. In order to reduce disease risk significantly, a reservoir population should be eliminated inside a 500-m radius of a protected area.[28] The difficulty of achieving such control renders the vector control option more attractive in many environments.

In some ways, phlebotomine vector control is very similar to that used for malaria; however, most larval control techniques used against mosquitoes are inappropriate for sand fly control because the aqueous habitat of the mosquito larva is very different from the highly organic soil requirement of the sand fly immature stages. One possible exception is the use of *Bacillus sphaericus* for sand fly larval control.[29] In this innovative technique, bait-fed adults were used to carry the bacterial control agent to larval habitats in animal burrows, resulting in larval mortality in burrows up to 10-30 m away from the baited solution.

The adult sand fly and the adult mosquito vectors, however, share characteristics that make control for one similar to that for the other. In fact, some researchers believe that one reason for the resurgence of leishmaniasis is a reduction in the use of insecticides for malaria control.[30–31]

Most adult sand fly control techniques can be placed in the following categories:

1. Residual sprays — the application of long-lasting insecticides to surfaces, often interior walls, to either kill or repel biting insects.
2. Space sprays — the dispersal of an insecticide in droplets or smoke to kill insects in the treated space, leaving little residual effect.
3. Barriers and treated netting/ clothing.
4. Topical repellents.
5. Applications in reservoir burrows.

Of these techniques, residual spraying of houses and animal shelters is probably the most useful and the most utilized. Several insecticides are available for potential use. A study of four species of sand flies from the Middle East indicated that DDT (an organochlorine) and malathion (an (organophosphate) exhibited less sand fly-specific toxicity than did the newer pyrethroid insecticides, including resmethrin and cyfluthrin.[32] DDT was the first insecticide used on a large scale for control of sand flies.[33] It was sprayed in large amounts in India, the Soviet Union, Brazil, China and elsewhere, causing noticeable reductions in sand fly populations. However, questions eventually arose as to whether the reduction in the number of insects was having a direct impact on disease risk. In 1991, R. P. Lane noted the following:

“Without doubt the single most important constraint on assessing the value of vector control in leishmaniasis control is the lack of well-documented examples of intervention; information is usually anecdotal and it is simply not possible to evaluate precisely the significance of sandfly control in disease control.“[34]

Some circumstantial evidence has been used to imply the effectiveness of sand fly control in reducing disease risk. One of the best examples is provided by the visceral leishmaniasis control program in Bihar, India, between 1958 and 1970. During these years, no visceral leishmaniasis cases were reported in Bihar, but the disease resurfaced soon after the cessation of the program.[33] Reductions in leishmaniasis were also noted with antimalaria programs that relied on interior residual sprays in Italy, Iran, Bangladesh and Peru. However, reductions in sand fly populations as a result of antimalaria programs did not reduce leishmaniasis disease rates in Greece or Portugal. These were among the first of several inconsistent results in the use of interior residual sprays that are reviewed in depth elsewhere.[33]

The switch from the organochlorines and organophosphates to the newer pyrethroid insecticides provided several new potential chemicals for use as residual sprays. In Brazil, deltamethrin was used for both interior and exterior applications. Sand fly populations inside houses were significantly reduced, but the exterior sprays were ineffective.[35] The long-term effect on morbidity due to leishmaniasis, however, was not assessed. Tests in other geographic areas often had similar results. In Kenya, where termite mounds and animal burrows were known sand fly habitats, a 90% reduction in sand fly populations was achieved following treatment of the mounds and burrows with a pyrethroid.[36] The reduction, unfortunately, only lasted for 2 weeks. In Israel, researchers attempted unsuccessfully to use cloths on which two insecticides (cyfluthrin and DDT) were applied to prevent sand flies from moving up a hill and into a village.[37] Conversely, a barrier of cyfluthrin on heavy woody undergrowth in Guatemala was effective at preventing sand flies from entering a cantonment inside the treated area and was effective for more than 80 days.[38]

The use of residual sprays is limited by several environmental factors, including high summer temperatures, strong radiation and the accumulation of dust.[37] These conditions can reduce insecticide toxicity or efficacy, and they have been especially important to the anti-leishmaniasis programs by American forces in Iraq. The risk of cutaneous leishmaniasis for American military personnel early in the conflict prompted intensive vector control efforts.[39] Nine different insecticides, including organophosphates, pyrethroids and a carbamate, were used as residual sprays, space sprays and dustings for animal burrows. Despite the use of a wide spectrum of techniques and chemicals, the program appeared to have limited success in reducing sand fly populations. Anecdotal accounts from soldiers, however, did indicate a reduction in sand fly bites after residual applications to tent walls. Paradoxically, some soldiers reported that biting increased after pesticide application, but then decreased significantly after 1 or 2 days.[40] The researchers speculated that the pesticides were initially irritants that caused the sand flies to increase biting rates. At present, this possibility has not been addressed.

It should be noted that the effectiveness of an interior residual spray may not be due to the lethal effects of the spray on the target vector population. Repellency and contact irritancy may be more important in protecting humans from exposure to vectors than is the lethal effect of the insecticide.[41] Such effects may be just as important with sand flies. The spatial repellency activities of other chemicals, including *neem* oil, citronella, linalool and geraniol, have also been tested against sand flies with varying levels of effectiveness.[42,43]

Space sprays have also been a part of some sand fly control programs. These sprays can be interior or exterior applications and do not leave a significant residual effect, being aimed at the insects in the environment at the time of the spray (usually the flying insects). As mentioned earlier, these sprays had disappointing effects in the U.S. military cantonments of Iraq.[39] Likewise, DDT and malathion fogs in Sudan were not effective in reducing sand fly populations, the results being both small and short-lived.[44] Nevertheless, interior fogs are still a part of some programs and can provide temporary relief from insect bites.

As with antimalaria efforts, the use of insecticide-treated nets (ITNs) has become an important part of many anti-leishmaniasis programs. These nets are very attractive because they can be effective, relatively cheap and sustainable. In addition, the pyrethroid insecticides used to treat the nets have relatively low mammalian toxicity and good insecticidal activity.[33] The inconsistent results obtained from interior residual sprays and the continued development of insect resistance to the insecticides of choice, including DDT, increase the importance of ITNs as sand fly control measures.[45] The nets can provide considerable protection when the local sand flies are endophagic (indoor feeding); however, the results of large-scale ITN programs run by charitable organizations have been inconsistent.[46] Trials in Iran and Syria had disappointing results. However, a significant reduction in visceral leishmaniasis was observed following the mass distribution of fine-mesh ITNs in Sudan.[47]

Variability in the effectiveness of ITNs is a result of numerous factors, including vector biology, the choice of insecticide/repellent, the net mesh size, net placement, and use compliance. In an early test, the topical repellent Deet (N,N-Deithy-m-toluamide) was applied to wide-mesh cotton netting and evaluated for protection against mosquitoes, phlebotomine sand flies and biting midges in Panama; the ITN provided effective repellency for 64 days.[48] Even untreated bed nets have proven effective at protecting humans from sand fly bites and are recommended as part of an anti-visceral leishmaniasis program in Nepal.[49] However, poor compliance with bed net usage is very common due to discomfort during hot weather. Work has been done with loosely hung ITN around windows and doors to prevent insect entry, and these have met with some success. Treated *chaddars* or top sheets have also been used successfully in Afghanistan.[50]

## THE FUTURE

Cutaneous leishmaniasis is considered by some to be a prime candidate for vaccine development.[46] A long history of “leishmanisation,” a process in which active infection is induced to produce natural “resistance,” suggests that an effective immunity can be developed. However, promising vaccines in murine models have been unsuccessful in primate and human trials.[51] The situation for visceral leishmaniasis is even less promising, but some recent progress has been made. Issues associated with vaccine development for leishmaniasis are reviewed elsewhere.[51–53] Until the implementation of an effective vaccine program, however, vector control will remain an important, if not the most important, disease-control strategy.

The HIV/AIDS pandemic may have an impact on the occurrence and presentation of leishmaniasis. Co-occurrence of the infections has led to the identification of leishmaniasis as a major opportunistic infection associated with AIDS in Brazil.[54] Diffuse cutaneous leishmaniasis, which may be confused with lepromatous leprosy, has been associated with HIV infection in India.[55] Although these issues may be of more interest to the clinician who is concerned with treating persons with co-infections, the situation is also of interest to those interested in disease prevention through vector control. In areas where visceral leishmaniasis is anthroponotic in transmission, co-infected patients may serve as potential reservoirs of infection.[56] Some additional efforts to prevent vector access to these patients may be required. A complete review of HIV/ leishmania co-infection is presented elsewhere.[56]

An important development in the epidemiology of leishmaniasis is the emergent “urban” form of the disease.[57] In the New World, urban leishmaniasis is usually associated with zoonotic cutaneous disease. The disease may be linked to remnants of rain forest and the presence of sylvatic reservoirs, including opossums and sloths. However, the documented spread of vectors and parasites into the intra-domiciliary and peri-urban environments may also play important roles. Urban visceral leishmaniasis has also been documented in Brazil.[58] In Asia, urbanized leishmaniasis is usually identified with anthroponotic transmission of cutaneous disease.

Leishmaniasis is a variable disease, reflecting the wide range of parasites in the genus *Leishmania*. Although progress has been made on the development of a vaccine, an effective one is not available. In the absence of a vaccine, disease prevention will continue to focus on vector control, augmented by other measures such as reservoir culling, screening and personal protection. The adaptability of the parasites and the vectors has allowed the disease to spread into new environments, including suburban and urban neighborhoods. These issues will require increased efforts in the fields of urban entomology, civil engineering, disease surveillance and tropical urban ecology in order to address the continued spread of this disease.
